# Autonomous Aerosol and Plasma Co‐Jet Printing of Metallic Devices at Ambient Temperature

**DOI:** 10.1002/smll.202409751

**Published:** 2025-02-16

**Authors:** Yipu Du, Jinyu Yang, Kaidong Song, Qiang Jiang, Md Omarsany Bappy, Yuchen Zhu, David B. Go, Yanliang Zhang

**Affiliations:** ^1^ Department of Aerospace and Mechanical Engineering University of Notre Dame Notre Dame IN 46556 USA; ^2^ Department of Chemical and Biomolecular Engineering University of Notre Dame Notre Dame IN 46556 USA

**Keywords:** aerosol and plasma co‐jet printing, ambient temperature processing, machine learning optimization, metal nanoparticles

## Abstract

Additive manufacturing of metallic materials holds the potential to revolutionize the fabrication of functional devices unattainable via traditional methods. Despite recent advancements, printing metallic materials typically requires thermal processing at elevated temperatures to form dense structures with desired properties, which presents a major challenge for direct printing and integration with temperature‐sensitive materials. Herein, a unique co‐jet printing (CJP) method is reported integrating an aerosol jet and a non‐thermal, atmospheric pressure plasma jet to enable concurrent aerosol deposition of metal nanoparticle inks and in situ sintering at ambient temperature. A machine learning algorithm is integrated with the CJP to perform real‐time defect detection and autonomous correction, enhancing the yield of printed films with high electrical conductivity from 44% to 94%. Concurrent printing and sintering eliminate the need for post‐printing processing, reducing the overall manufacturing time by multiple folds depending on product size. CJP enables direct printing of functional devices on a variety of temperature‐sensitive materials including biological materials. Direct printing of hydration sensors on living plant leaves is demonstrated for long‐duration monitoring of hydration level in the plant. The versatile CJP method opens tremendous opportunities to harmoniously integrate abiotic and biotic materials for emerging applications in wearable/implantable devices and biohybrid systems.

## Graphical abstract

This study presents an aerosol and plasma co‐jet printing technique capable of concurrently depositing metal nanoparticle inks and performing in situ plasma jet sintering at ambient temperature, eliminating the need for prolonged high‐temperature post‐processing. A machine learning algorithm is employed to detect printing defects and improve manufacturing yield. Using this method, a silver nanoparticle‐based humidity sensor is directly fabricated on an English Ivy leaf, successfully detecting the plant's hydration level changes over more than 30 hours.

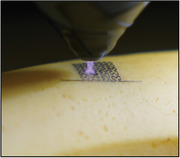

## 1 Introduction

Additive manufacturing (AM) is revolutionizing the fabrication of electronic devices by enhancing design freedom and accelerating the development cycle, which paves the way for cutting‐edge technologies in flexible/wearable electronics,^[^ ^1^, ^2^, ^3^, ^4^ ^]^ human‐machine interfaces,^[^ ^5^ ^]^ the Internet of Things,^[^ ^6^ ^]^ and various other fields. The emergence of aerosol‐based printing of functional devices has showcased distinct benefits, including the production of ultra‐fine features,^[^ ^7^, ^8^, ^9^, ^10^, ^11^ ^]^ the versatility in processing a broad spectrum of materials,^[^ ^12^, ^13^, ^14^, ^15^ ^]^ and the capability for conformal printing and combinatorial printing.^[^ ^16^, ^17^, ^18^, ^19^, ^20^ ^]^ These attributes enable the construction of a diverse array of functional devices with unique properties that are unattainable through traditional methods.^[^ ^5^, ^21^, ^22^, ^23^, ^24^, ^25^, ^26^, ^27^, ^28^ ^]^ Nonetheless, similar to other printing techniques that employ metallic or semiconducting nanoparticle inks, aerosol‐based printing typically requires a post‐printing sintering process to remove surfactants and binders and promote atomic diffusion in order to form a dense structure with desired electrical conductivity.^[^ ^20^, ^29^, ^30^, ^31^ ^]^ This not only takes considerable time but also demands thermal processing at elevated temperatures, which present a major barrier for printing metallic devices on temperature‐sensitive substrates such as biological materials, and multi‐materials printing to integrate inorganic and organic materials.

To facilitate rapid sintering and remedy the temperature restrictions, various alternative sintering techniques have been explored. Flash sintering, for instance, employs intense light pulses to sinter the deposited material while minimizing the thermal effect on the underlying substrate.^[^ ^29^, ^32^, ^33^, ^34^ ^]^ However, it is still an inherently thermally driven process and necessitates heating of the printed materials to a certain temperature and additional processing time. Chemical sintering, on the other hand, involves in situ chemical reactions to achieve sintering of printed nanoparticles at low temperatures, but is restricted to specific inks, limiting potential substrates, and requires post‐sintering cleaning of the films.^[^ ^35^, ^36^ ^]^

Non‐thermal plasma sintering is an attractive alternative that has gained increasing interest in recent years. A non‐thermal plasma is a partially ionized gas consisting of highly energetic electrons (≈1–10 eV or ≈10**^5^** K), while the ions and neutral species are not in thermal equilibrium with these electrons, resulting in an overall low gas temperature that can remain close to ambient temperature.^[^ ^37^, ^38^ ^]^ Therefore, the promotion of sintering is hypothesized to be primarily driven by energetic, chemically reactive plasma species and/or photons, rather than by thermal effects.^[^ ^39^, ^40^ ^]^ To date, a variety of non‐thermal plasma configurations have been implemented in sintering, including low pressure (mbar) radio frequency (RF) plasmas,^[^ ^40^, ^41^, ^42^, ^43^ ^]^ atmospheric pressure dielectric barrier discharge (DBD) plasmas,^[^ ^44^ ^]^ and atmospheric pressure plasma jets (APPJs). APPJs utilize a working gas flow to extend the ionization zone and propogate the plasma to a remote distance with a confined shape, allowing indirect and non‐proximal plasma sintering. APPJs have been explored for processing a diverse array of metallic and polymer‐based materials.^[^ ^39^, ^44^, ^45^, ^46^, ^47^, ^48^, ^49^, ^50^, ^51^, ^52^, ^53^, ^54^, ^55^, ^56^, ^57^, ^58^ ^]^ The utilization of a working gas flow in an APPJ makes it well‐suited for integration with aerosol jet printing to enable both in situ and low temperature sintering of metallic inks at the point‐of‐printing, which significantly reduces the time required for post‐printing treatments. However, current configurations of plasma jet printing primarily rely on directly introducing aerosol droplets into plasma jet flow.^[^ ^47^, ^48^, ^49^ ^]^ The intricate interactions between plasma and aerosol droplets, along with the lack of dedicated mechanisms for high‐resolution aerosol deposition, pose challenges in achieving effective sintering while maintaining printing quality. Moreover, a relatively high voltage of over 5 kV is usually required to initiate the plasma jet and additional insulation is necessary to prevent gas breakdown outside of the aerosol flow, which further constrains the design of the plasma reactor.

**Figure 1:**
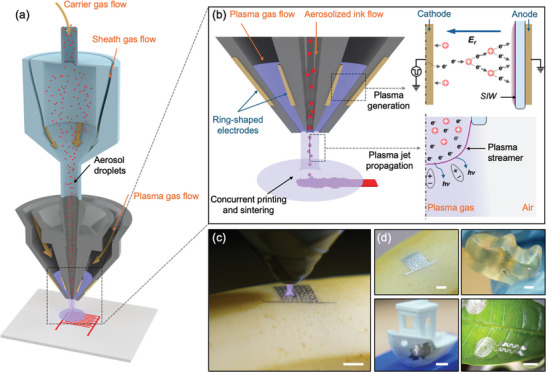
Configuration and principles of the aerosol and plasma co‐jet printing (CJP) system. a) Schematic of the CJP consisting of an aerosol jet and a coaxial atmospheric pressure plasma jet (APPJ) for concurrent ink deposition and sintering. b) The annular plasma jet flowing coaxially surrounding the aerosol ink flow through the outer path of the deposition nozzle. The plasma is generated between two ring‐shaped electrodes and continues to propagate after exiting the nozzle. c) Active CJP of silver nanoparticle ink on the surface of a banana. d) Conductive silver patterns directly printed on a banana (top left), a gummy bear (top right), a benchmark boat printed using polylactic acid (bottom left), and a leaf (bottom right). Scale bars for (c,d) are 5 mm.

Herein, we report a unique co‐jet printing (CJP) method that integrates an ink‐containing aerosol jet and a non‐thermal atmospheric‐pressure plasma jet in a coaxial configuration, enabling direct and concurrent deposition and sintering of printed materials with high resolution and conductivity on delicate substrates, such as natural biological materials and soft polymers (**Figure** **1**). To achieve high reproducibility and yield, a machine learning‐enhanced real‐time process monitoring and control algorithm is developed to realize autonomous online defect detection and compensation. The CJP delivers a competitive electrical conductivity in printed silver films comparable to ex situ thermal sintering techniques while maintaining the processing temperature near ambient, reducing the overall manufacturing time by multiple folds by eliminating the need for post‐printing thermal sintering. We further demonstrate direct printing of hydration sensors on living plant leaves for long‐duration monitoring of hydration level changes in the plant.

## 2 Results and Discussion

### 2.1 Design and Principle of the Co‐Jet Printing System

The CJP system consists of two primary components, as shown in Figure 1a. The first is responsible for the generation, transportation, and deposition of aerosolized inks, which has the same setup as in our previous work.^[^ ^17^ ^]^ The prepared ink solution undergoes ultrasonic atomization to form aerosolized droplets, which are then transported to the deposition nozzle by an inert carrier gas flow. At the nozzle, the aerosol is aerodynamically focused by an annular sheath gas flow to form a narrow stream aerosol jet, enabling precise deposition of high spatial resolution patterns onto the substrate.^[^ ^59^, ^60^ ^]^ The second component responsible for plasma jet generation takes the form of an annular pulsed DBD plasma jet with the plasma working gas (hereafter referred to as plasma gas) flowing coaxially to the aerosol jet through the outer (annular) path of the deposition nozzle, as shown in Figure 1b. A cathode electrode is mounted on the inner nozzle wall and a grounded anode electrode is mounted on the outer nozzle wall. The inserts in the figure illustrate the fundamental processes of plasma jet formation and propagation. In short, as the plasma is formed between the two electrodes, the plasma gas propagates the plasma through the exit of the nozzle, where it meets the aerosol jet. (More details on this process can be found in Figure S1, Supporting Information). To that end, the generated plasma jet interacts with the aerosols both during flight and as they impact and spread on the substrate, simultaneously sintering the printed nanoparticles during deposition. Furthermore, the ambient temperature nature of the APPJ allows for printing on delicate substrates. Another advantage of this design is the decoupling of the aerosol gas flow and the plasma gas flow, which allows fine tuning of different gas combinations and flow rates for different substrate materials, inks, and devices. The direct printing of conductive silver patterns on a wide range of substrates is demonstrated, including a leaf, a banana, gelatin, polylactic acid, and Ecoflex^TM^ silicone rubber (Figure 1c; Figure S2, Supporting Information).

### 2.2 Characterization of the Co‐Jet Printing System

**Figure 2:**
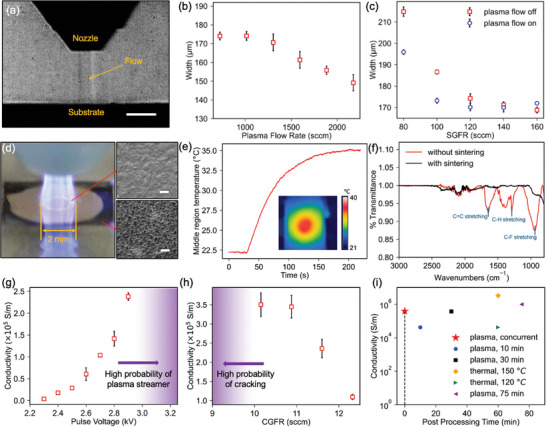
Characterization of the CJP system. a) A Schlieren image shows the density gradients of the gases emitted from the nozzle. Scale bar is 4 mm. b) The printed line width versus the plasma gas flow rate at a constant sheath gas flow rate (SGFR) (*n* = 5). c) The printed line width versus the SGFR with and without the plasma gas flow (*n* = 5). d) The shape of the plasma jet and SEM images of the profile of the plasma sintering effect both within a 1 mm radius of the plasma impingement point (top) and at roughly a 3 mm radius outside the impingement point (bottom). Scale bars are 10 µm. e) The temperature change in the region impacted by the plasma jet during a continuous exposure of 200 s. f) FTIR results of the unsintered and CJP printed samples, show the removal of organic components associated with the surfactant in the ink. g) The conductivity of the film versus the plasma pulse voltage (*n* = 3). h) The conductivity of the film versus the carrier gas flow rate (CGFR) (*n* = 3). The error bars in (g,h) represent the random errors in conductivity of films manufactured under identical printing and sintering parameters. The relatively small errors indicate that the performance of the CJP method is stable and repeatable. i) The conductivity versus post‐processing time for the silver films printed by CJP and by other methods.^[^ ^39^ ^]^

The CJP printing head features an inner nozzle for delivering the aerosol ink and sheath gas flows, along with an outer annular‐shaped component to deliver the plasma gas flow. **Figure** **2**a shows the co‐jet flow exiting the nozzle using Schlieren imaging (Figure S3a, Supporting Information). The combined stream of the carrier gas flow, sheath flow, and plasma gas flow remains laminar, with minimal interference between the inner and outer flows. These properties persist over 10 mm, significantly greater than the typical nozzle‐to‐substrate working distance (≈1–5 mm), allowing for simultaneous printing and sintering without compromising the printing quality. The optimal operating window of the nozzle used in this work for maintaining laminar flow has been characterized using Schlieren imaging and is presented in Figure S3 (Supporting Information).

Previous studies have demonstrated the focusing effect of the sheath gas flow on reducing the width of a printed line during aerosol jet printing.^[^ ^17^, ^59^, ^60^, ^61^ ^]^ In this work, the introduction of the plasma gas flow preserves this characteristic of aerodynamic focusing. As shown in Figure 2b, an increase in the plasma gas flow further reduces the line width at a constant sheath gas flow rate (SGFR), thereby slightly enhancing the resolution of deposition on a glass substrate. Figure 2c shows the printed line width under various SGFR with and without a coaxial plasma gas flow. Notably, at lower SGFR, including a plasma gas flow results in narrower line widths compared to those printed with only sheath gas. At higher SGFR, the line widths converge to those obtained with sheath gas alone.

After impinging on the glass substrate, the plasma jet induces radially propagating surface ionization waves (SIWs) along the surface of the substrate due to the charging of the dielectric material, thereby creating a circular region where sintering of the printed materials may occur. To evaluate the region of effective sintering, morphology changes before and after plasma jet sintering were characterized using scanning electron microscopy (SEM) at various sites across a 6 mm by 6 mm film. This film was printed with silver nanoparticle ink and post‐sintered using the plasma jet positioned stationarily above the film center, such that only the area of plasma‐film contact could be sintered. As shown in Figure 2d, the most significant sintering occurred directly at the plasma‐substrate impingement point, within a circular region of ≈2 mm in diameter. However, owing to SIWs on the substrate, the sintering effect extends up to a diameter of roughly 6 mm, though the visual quality of the sintering decreases further from the impingement point. Importantly, this reveals that both the aerosols currently being deposited and those previously deposited in the adjacent regions undergo sintering with this CJP system, which leads to the increased overall exposure time to the plasma jet to increase sintering efficacy.

Time‐resolved infrared (IR) camera measurements were conducted to monitor the glass substrate temperature during the CJP process. As shown in Figure 2e, the average temperature of the region processed by the plasma jet gradually increases from room temperature to ≈35 °C in 2 min and then reaches a plateau due to the balance of energy input and dissipation. While this temperature rise varies depending on the substrate material, our experiments with organic and biological substrates, as shown in Figures 1c,d and S2 (Supporting Information), indicate that the surface temperature remains below the safe threshold for most thermal‐sensitive substrate materials, which confirms the ambient temperature nature of the CJP system. Even without a significant rise in temperature, the energetic plasma species are still able to remove the electrically insulating polymeric surfactants in the ink from the printed nanoparticles and promote sintering. As shown in Fourier‐transform infrared spectroscopy (FTIR) measurements in Figure 2f, the absorption peak signatures for the surfactant, such as the C═C stretching band at 1645 cm^−1^, C─H stretching band at 1288 cm^−1^, and C‐F stretching band at 940 cm^−1^, disappeared when using the CJP approach, demonstrating the effective removal of surfactant from the printed silver film.^[^ ^62^, ^63^ ^]^

The conductivity of a film printed using CJP is influenced by various processing parameters. Among these, the pulse voltage of the plasma jet plays a critical role, affecting the intensity of the plasma jet and the energy carried by it. As shown in Figure 2g, increasing the pulse voltage from 2.3 to 2.9 kV enhances the conductivity of the printed films by a factor of ≈100, when all other parameters are fixed. However, increasing the pulse voltage beyond 2.9 kV often led to the formation of a single strong streamer due to the generation of an excessive number of charged species at the high applied electric field.^[^ ^64^, ^65^ ^]^ The plasma then tends to thermalize and increase in temperature, which significantly increases the risk of nozzle material breakdown and potential damage to the printed film.

When plasmas are used for sintering ex situ after printing, the effective sintering depth has been reported to extend only a few nanoparticle layers beneath the plasma‐surface contact, leaving the bottom layers unsintered.^[^ ^40^, ^41^ ^]^ This inhomogeneity has been hypothesized to result from the formation of a dense, continuous shell that prevents the penetration of reactive plasma species and hinders the escape of volatile species from the bulk.^[^ ^41^, ^66^ ^]^ Therefore, a low‐to‐medium material deposition rate during CJP is favorable as it results in thinner layers that are more effectively treated by the plasma in situ during each pass. As depicted in Figure 2h, reducing the aerosol carrier gas flow rate (CGFR) from 12.3 to 10.2 sccm results in an approximate 3.5‐fold increase in conductivity. Further decreasing the CGFR leads to inadequate material deposition, which produces defects such as voids on the film. Charged plasma species are attracted to the edges of the voids due to a locally enhanced electric field, causing them to be etched and eventually connected to form cracks (Figure S4, Supporting Information). As shown in Figure 2i, the CJP method achieves conductivity comparable to that of an equivalent post‐printing plasma sintering process conducted for 30 min and even surpasses the conductivity obtained through thermal sintering at 120 °C for 60 min. Since the CJP method eliminates the need for post‐processing, it has the potential to significantly enhance the overall manufacturing efficiency, reducing the time by several folds depending on the product size, as demonstrated in Figure S5 (Supporting Information).

### 2.3 Machine Learning‐Enabled In Situ Defect Detection and Compensation

**Figure 3:**
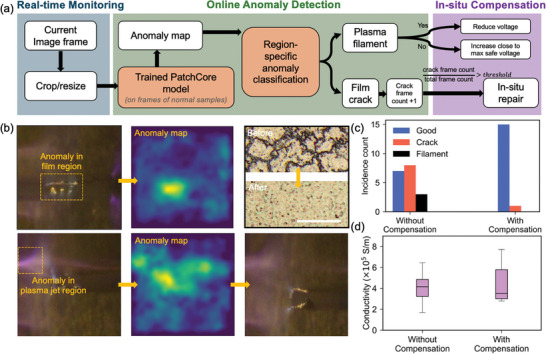
In situ defect detection and compensation. a) Machine learning‐enabled real‐time monitoring process for region‐specific anomaly detection and compensation. b) Frames that identify anomaly. The top frames show detected cracks, and the bottom frames identify a plasma filament. Scale bar is 10 µm. c) The improvement in yield with in situ defect detection and compensation. d) The conductivity comparison of good silver films printed without and with in situ compensation.

CJP is a complex process due to the intricate interplay between the material deposition and plasma jet sintering processes, as well as potential instability of the plasma jet. Consequently, there is often a trade‐off between increasing conductivity and reducing the defect rate thus improving the yield. For example, increased plasma voltage improves conductivity but also increases the probability of plasma streamer formation and thus film damage. To address these challenges, we incorporated a camera for real‐time monitoring along with a region‐specific anomaly detection algorithm into the CJP system, as shown in **Figure** **3**a. This setup enables the real‐time identification and analysis of anomalies such as filamentation in the plasma leading to streamer formation and film cracking, which are incorporated into a control algorithm for immediate adjustment and compensation based on the feedback received.

The region‐specific anomaly detection algorithm utilized in our system leverages frame‐by‐frame analysis using the PatchCore machine learning model with the unique ability to detect subtle anomalies rapidly by relying solely on nominal images for training.^[^ ^67^ ^]^ The effectiveness of the model is achieved upon the curation of a maximally representative memory bank of nominal patch features extracted from a pre‐trained convolutional neural network (CNN). Additionally, the coreset sampling technique is applied to down sample the memory bank. The training process is detailed in the experimental section. During inference, the test patch features were extracted from the same pre‐trained CNN. The pixel level anomaly score is determined by the nearest neighbor distance between the corresponding test patch feature and those within the memory bank. Given that filamentation typically occurs in the plasma jet region, the image is segmented into the plasma jet region and printed film region, and the occurrence of filamentation and film cracking is determined by the pixel level anomaly score within each region.

During printing, the trained PatchCore model is integrated into the camera monitoring program, continuously providing an anomaly map for each live frame, where each pixel has a value indicating the pixel level anomaly score of the current frame every second (Figure 3a). If an anomaly is detected in the region of plasma jet sintering, the pulse voltage of the plasma jet is promptly reduced by 0.5 kV. If no anomaly is detected, the voltage incrementally increases by 0.1 kV until an anomaly is observed, after which it is set to 0.2 kV below that level. When anomalies are detected in the printed film region, the current frame is marked as anomalous, and the ratio of anomaly frame count to the total frame count is calculated for each printing pass. If this anomaly ratio exceeds a predefined threshold, indicating a cracked layer, a compensation mechanism is activated to repair it by printing a new layer without active plasma jet, followed by another layer with concurrent printing and sintering. The first row of Figure 3b displays a captured frame indicating a suspected crack. The edges of the holes on the film increase the local electric field and result in the formation of plasma from the edges, which the algorithm detects as an anomaly, as shown in the anomaly map. After the anomaly was detected, the compensation mechanism was able to repair the defect. The optical microscope images illustrate the presence of a crack without compensation and a smooth film after compensation. The second row presents a frame with detected plasma streamer, appearing as an anomaly in the plasma jet region on the anomaly map. Adjustments to the pulse voltage successfully eliminate the filament.

The in situ monitoring and correction method significantly enhances the yield and reproducibility of the CJP process. We printed 16 samples with parameters randomly sampled from a range considered prone to defects (CGFR between 8 and 10 sccm, plasma pulse voltage between 2.8 and 3 kV, Figure S6, Supporting Information). The atomizer voltage was randomly selected from 30 to 40 V to manually introduce system drifting effects, which is considered as the main cause of process instability in aerosol jet printing.^[^ ^59^ ^]^ As demonstrated in Figure 3c, without the use of in situ monitoring, 8 out of 16 samples exhibited cracks, and streamer formation was observed in 3 samples. However, the yield of defect‐free samples increased to 94% with in situ monitoring and correction under the same set of parameters, with only one cracked sample remaining undetected and uncompensated. Furthermore, the conductivity ranges of defect‐free samples and the samples subjected to in situ compensation are presented in Figure 3d. The conductivity of the samples with in situ corrections is comparable to that of samples without defects and corrections, suggesting that the applied compensation does not substantially affect the conductivity of the printed samples.

### 2.4 Applications and Demonstration of Co‐Jet Printing

The capacity for simultaneous printing and sintering near ambient temperature positions the CJP system to overcome various limitations faced by conventional AM methods, thereby facilitating broad applications across diverse fields. One of the major trends in AM is multi‐material and multi‐scale fabrication, which is often hindered by fundamental material and process incompatibility between functional inorganic materials and organic materials.^[^ ^2^, ^4^, ^23^, ^24^, ^68^, ^69^ ^]^ Here we demonstrate that CJP enables direct printing of functional devices on biological interfaces, such as animal skin and plant epidermis.

**Figure 4:**
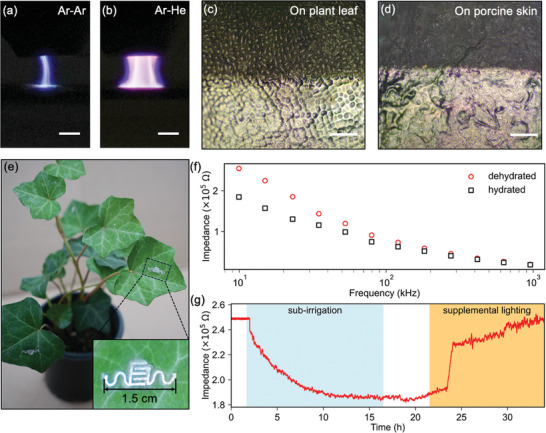
Demonstration of CJP of silver films and sensors on biomaterials. Photograph of the plasma jet with a) argon as both the aerosol sheath gas and plasma gas, and b) argon as the aerosol sheath gas and helium as the plasma gas when printing on a leaf. Scale bars are 2 mm. Microscope images of printed silver films on c) a plant leaf and d) porcine skin. Scale bars are 200 µm. The top half is the substrate without silver and the bottom half is covered with printed silver. e) The image of a pot of English ivy with hydration sensors directly printed on its leaves. The inset shows a detailed view of the printed interdigitated silver electrode. f) The impedance of hydrated and dehydrated leaves measured under different frequencies. g) The detected impedance shift of the printed sensor at 10 kHz under various controlled environmental changes affecting the hydration level of the leaf.

The decoupling of aerosol and plasma gas flows in the CJP system enables precise control over gas composition and flow rates, allowing for fine‐tuning to accommodate various substrates, inks, and devices. Initial development as described in Sections 2.2–2.3 utilized low‐cost argon gas as both the aerosol sheath gas and the plasma gas. However, when printing on a naturally conductive substrate, such as a water‐containing biological interface, the plasma jet tends to filament into an attached streamer.^[^ ^70^, ^71^, ^72^ ^]^ In order to overcome this issue for practical application, we switched the plasma gas to helium while maintaining argon as the aerosol sheath gas, which led to a stable, glow‐like plasma jet even on a water‐containing biological interface (a living plant leaf), as shown in **Figures** **4**a,b. Under these conditions, the electrical conductivity of the printed silver electrode reaches 1.2 × 10^6^ S m^−1^, which is greater than the values obtained on polymer and glass substrates. This enhanced conductivity is primarily attributed to the conductive nature of the biological materials, which facilitates greater current flow through the plasma, leading to a higher density of reactive plasma species that further promote the sintering of the printed nanoparticles.^[^ ^73^, ^74^ ^]^ In addition, the porosity and morphological characteristics on top of the biological interface also influence the plasma behavior^[^ ^75^, ^76^, ^77^, ^78^, ^79^ ^]^ and its interaction with the printed silver nanoparticle ink,^[^ ^39^ ^]^ ultimately affecting the efficiency of plasma sintering.

The advanced direct fabrication of nanoscale materials on biological interfaces via CJP offers numerous advantages over the traditional transfer‐after‐fabrication approach. First, the printed materials achieve unparalleled 3D conformity and intimate contact and coupling with the biological materials, as the nanoparticles are despersed in liquid solution and the electrodes are formed in place on the biological materials. Figures 4c,d present optical microscope images of the printed silver films on both leaf epidermis and porcine skin, where the microstructures of the biomaterials are precisely replicated by the overlaying electrodes, demonstrating their truly conformal nature. This property is crucial for applications that require low contact resistance or effective strain transfer.^[^ ^80^, ^81^, ^82^ ^]^ Second, it allows for the minimization of device weight and size, reducing the impact on the normal activities of the plants or animals.^[^ ^83^, ^84^, ^85^ ^]^ Third, the approach enhances the level of automation in the manufacturing process, which eliminates errors associated with human operation and thereby increases the repeatability and yield of the manufacturing process.

To demonstrate the direct printing on biological interfaces, a sensor designed to measure the hydration level in plant leaves was directly printed on the leaves of a potted English ivy plant, as depicted in Figure 4e. Due to the intimate contact of the sensor and leaf epidermis, the sensor, in the form of an interdigitated electrode pair, is able to measure the hydration level via an electrochemical impedance measurement.^[^ ^86^, ^87^ ^]^ Figure 4f shows impedance measurements under various frequencies in both dehydrated (dry) and hydrated (watered) states of the plant. The difference between the measured signals is more pronounced at lower frequencies, indicating enhanced sensitivity within this range. A frequency of 10 kHz was selected to measure the impedance of the sensor in response to different events that impact hydration level of the leaf over time, as shown in Figure 4g. The potted English ivy was subjected to a 24‐h dehydration period to reach a dried condition. Subsequently, the plant was hydrated in a simple sub‐irrigation system for ≈15 h. During this period, a gradual decrease in impedance was observed due to water absorption through transpiration. After reaching a plateau, the plant pot was removed from the sub‐irrigation reservoir and the impedance started to increase after a short period of time. Following this, supplemental LED lighting was introduced to enhance photosynthesis, and a rapid increase of impedance was observed due to the significant consumption of water storage in the leaf. The impedance reverted to the previous level of the dried plant after 12.5 h. The detected hydration level changes not only demonstrate the efficacy of the sensor fabricated by the CJP but also suggest minimal disturbance of CJP on the plant cells.

## 3 Conclusion

Integrated aerosol and plasma CJP for simultaneous printing and sintering metallic nanomaterials at atmospheric pressure and ambient temperature delivers a high conductivity comparable to conventional aerosol jet printed materials that require extended post‐printing sintering at elevated temperature. A machine learning‐directed in situ defect detection and compensation method for the CJP significantly improves the process reproducibility and yield. A hydration sensor directly printed on a living English ivy plant accurately monitors the change of hydration levels with minimal impact on the plant. The CJP opens up numerous opportunities to harmoniously integrate abiotic and biotic materials, which has significant impact on the development of wearable/implantable devices for personalized healthcare, human‐machine interfaces, and biohybrid systems. To further enhance the capability of the CJP method and enable concurrent printing and sintering on various irregular surfaces, 6‐axis printers could be developed to ensure the printing head remains perpendicular to the substrate and maintains a constant nozzle‐to‐substrate distance.

## 4 Experimental Section

### Materials

A 12% w/w aqueous silver nanoparticle ink was employed in this work, which consisted of a 50% w/w commercial silver nanoparticle ink (JS‐A221AE, Novacentrix) diluted with deionized (DI) water. Additionally, the ink formulation included 6% w/w ethylene glycol (EG). 1.2 mL of prepared ink was used for each printing task, and 9 µL defoamer (Five Star defoamer 105) was added to avoid bubbling during atomization.

### Fabrication of the CJP Nozzle

The inner and outer co‐jet nozzle parts were fully printed with resin (ELEGOO Standard 8K 3D Printer Resin) using a stereolithography printer. A brass sleeve (7473T192, McMaster) was installed on the inner nozzle and served as the inner working electrode. A copper wire (7940A91, McMaster) was wrapped around the outer nozzle and served as the outer ground electrode. The inner and outer nozzles were assembled and sealed with epoxy. The diameters of the inner and the outer nozzle are 540 µm and 2.4 mm, respectively. The smallest inner nozzle diameter that can be fabricated by our current stereolithography printer is 260 µm, yielding a minimum line width of ≈60 µm as shown in Figure S7 (Supporting Information).

### Plasma Jet Generation

The plasma gas flowed through the outer path of the nozzle at the desired flow rate and was metered by a mass flow controller (Omega, FMA5518A). A direct current power supply (Matsusada Precision Inc., AU‐5R120) provided power to a pulse generator (Berkeley Nucleonics Corp., PVX‐4140‐B) between −3.2 and 0 kV. Unless otherwise stated, the plasma pulse voltage was maintained at −2.9 kV. The pulse generator was gated with a 5 V square waveform signal at a frequency of 20 kHz and a duty cycle of 30%, produced by a function generator (RIGOL, DG4062), for all experiments. The electrical characteristics of the plasma jet were monitored using a digital oscilloscope (RIGOL, DS4024).

### Schlieren Imaging

The co‐jet flow existing the nozzle was visualized using Schlieren imaging. Laser beams (CAVILUX^®^ Smart, 640 nm) were collimated to illuminate the interface of the co‐jet flow and the glass substrate. Beams deflected by the flow had a chance of bypassing a knife edge (FatMax Blade, 11–700A) and were captured by a high‐speed camera (Photron FASTCAM, SA4) aligned with the substrate surface at an imaging rate of 10^3^ fps, while undisturbed beams were intercepted by the knife edge. The resulting image, as illustrated in Figure 2a, showed variation in light intensity, manifesting the changes in the refractive index caused by density gradients within the flow. A schematic of the Schlieren imaging setup is provided in Figure S3 (Supporting Information).

### Characterization of the CJP Process

Characterization of the CJP process, as presented in Figure 2, was conducted on glass substrates (MSL3×1, United Scientific) that were cleaned with isopropyl alcohol prior to deposition. Silver nanoparticle ink was used for all experiments, with all performed under a constant printing speed at 1 mm s^−1^, atomizer voltage at 30 V, ink bath temperature at 22 °C, plasma pulse frequency of 20 kHz and plasma pulse duty cycle of 30%. Argon was employed as both the plasma gas and the carrier/sheath gas for the aerosol flow. The impact of plasma gas flow on the line width was measured with SGFR maintained at 120 sccm and CGFR at 8.7 sccm. The impact of SGFR on the line width was measured with plasma gas flow maintained at 1000 sccm and CGFR at 8.7 sccm. 4 passes were printed to form the lines. The plasma pulse voltage was −2.7 kV to initiate the plasma jet. The impact of plasma pulse voltage on conductivity was performed under constant SGFR at 120 sccm, plasma gas flow at 1300 sccm, and CGFR at 8.7 sccm. The impact of CGFR on conductivity was performed under constant SGFR at 120 sccm, plasma flow at 1300 sccm, and plasma pulse voltage at −2.9 kV. The thickness of the fabricated film varies depending on the printing and sintering parameters, as is presented in Table S1 (Supporting Information).

Printed pattern resistances were measured with a custom 4‐probe measurement platform (349701A, Keysight and DP281, RIGOL). Three layers were printed to form the films. The total thickness of the printed pattern was measured with a white light interferometry profilometer (Profilm3D, Filmetrics). Scanning electron microscope (SEM) images were acquired with Helios G4 UC Dual Beam, and optical microscope images with Olympus BX53M. FTIR measurements were conducted with Shimadzu IRXross. Data are presented as the mean with the error bars representing the standard deviation. A sample size of *n* = 5 was used for Figures 2b,c, and a sample size of *n* = 3 was used for Figures 2g,h.

### Thermal Characterization

An IR camera (FLIR T420) was utilized to measure the spatiotemporal evolution of the temperature distribution on the glass substrate. A schematic of the experimental apparatus is shown in Figure S8 (Supporting Information). Owing to the unknown emissivity of the glass substrate, and to avoid possible interference from IR radiation emitted by the plasma, 0.2 mm thick black electrical tape with an emissivity of 0.92 was placed on the backside of the substrate. The IR camera was then positioned 0.2 m away, directed at the backside of the substrate. Our previous work showed that the temperature difference between the front and back surfaces of the substrate was within the measurement uncertainty of the IR camera,^[^ ^39^ ^]^ indicating that the back‐surface temperatures can effectively approximate those at the front surface. IR measurements were collected for all characterization experiments at a rate of 30 fps and temperatures presented in Figure 2 have an uncertainty of ±2 °C.

### In Situ Detection and Compensation

The in situ detection and compensation system consisted of an inspection camera (Dino‐Lite Premier) placed above the substrate (Figure S9, Supporting Information), recording live images at a frame rate of 1 fps. Images were recorded with a PC running a control system using Python with an integrated PatchCore machine learning algorithm.

The PatchCore model was trained using 18 samples printed with randomly generated printing and plasma jet parameters and the processes were recorded with the inspection camera for data collection. First, 200 frames randomly collected from the printing processes of defect‐free films were input into a pre‐trained WideResNet‐50. The intermediate level features were extracted from the network and aggregated to form a memory bank. Then, coreset subsampling with a 5% sampling rate was performed on the memory bank to preserve the most critical information for future computation while decreasing inference time and memory usage. During inference, the test patch features were extracted in a manner identical to the training phase using WideResNet‐50. Anomalib1.0 was used for the training of the PatchCore model.^[^ ^88^ ^]^

The validation set consists of 10 video frames from the manufacturing process of anomaly‐free samples, 10 manually selected video frames showing a strong single streamer, and 10 video frames showing cracks. The threshold for identifying film cracking‐related anomalies was determined by validating on a set of 10 labeled frames associated with anomaly‐free films and 10 frames associated with cracked films. Validation was performed using the functions in Anomalilab1.0, which maximize the model's prediction accuracy on the validation set. Similarly, the threshold for identifying a plasma filamentation‐related anomaly was determined by validation on a set of 10 labeled frames associated with normal films and 10 frames associated with strong single streamers. To enhance robustness, whether the printed layer is classified as cracked or not is determined by the ratio of crack‐suspected frames to the total number of frames for that layer. The threshold of this ratio was optimized to maximize the model's prediction accuracy, measured by the F1 Score, on all collected videos used for training.

### Co‐Jet Printing of the Hydration Sensor Hydration Measurements

A pot of English Ivy was placed on an electrically insulated printing platform. A leaf was secured on a flat surface to avoid undesired movement. The hydration sensor was printed using the CJP on the leaf with an argon SGFR of 120 sccm, an argon CGFR of 10 sccm, a helium plasma gas flow of 1800 sccm, and a plasma pulse voltage at 2.2 kV. The printing speed was 1 mm s^−1^ and the atomization voltage was 30 V; the electrode pattern was printed with three passes. No post‐processing was conducted.

The hydration sensor signal using impedance spectroscopy, was measured by an electrochemical workstation (Interface 1010E). The leaf with the printed hydration sensor was placed on an elevated flat surface, with its edges taped to minimize potential motion. Copper wires were bonded to the wiring pads of the printed sensor using silver paste and were secured to the flat surface with tape to prevent movement during testing. For the transient measurement in Figure 4g, the impedance was monitored continuously using a measurement frequency of 10 kHz. A Savitzky–Golay filter was applied to smooth the measured signal. A cold LED lamp (ES250UFO, Hytekgro) was placed above the plant to act as a supplemental light source and stimulate photosynthesis.

## Conflict of Interest

The authors declare no conflict of interest.

## Supporting information



Supporting Information

## Data Availability

The data that support the findings of this study are available from the corresponding author upon reasonable request.
