# Supplementary material for: Autonomous Aerosol and Plasma Co‐Jet Printing of Metallic Devices at Ambient Temperature
Source: Small. 2025 Feb 16;21(11):2409751. doi: 10.1002/smll.202409751 (PMC11922012; doi:10.1002/smll.202409751)
Supplement: Supplementary file 1 — Supporting Information [file SMLL-21-2409751-s001.docx]

Supporting Information

Autonomous Aerosol and Plasma Co-Jet Printing of Metallic Devices at Ambient Temperature

*Yipu Du, Jinyu Yang, Kaidong Song, Qiang Jiang,* *Md Omarsany Bappy, Yuchen Zhu, David B. Go^*^, Yanliang Zhang^*^*

Y. Du, J. Yang, K. Song, Q. Jiang, Y. Zhu, D. B. Go, Y. Zhang

Department of Aerospace and Mechanical Engineering, University of Notre Dame, Notre Dame, IN 46556, USA

E-mail: [dgo@nd.edu](mailto:dgo@nd.edu), [yzhang45@nd.edu](mailto:yzhang45@nd.edu)

D. B. Go

Department of Chemical and Biomolecular Engineering, University of Notre Dame, Notre Dame, IN 46556, USA

*Yipu Du and Jinyu Yang contributed equally to this work.*


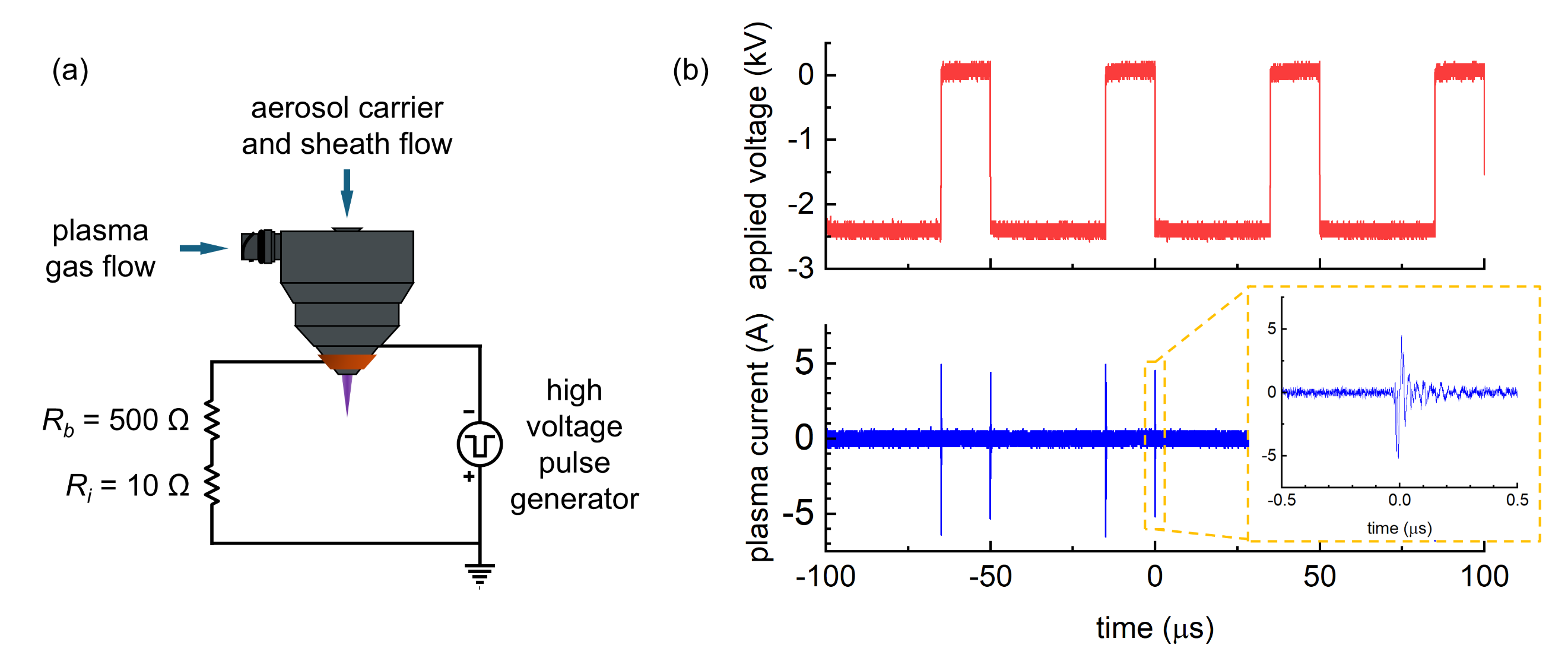


Figure S1: (a) Schematic illustration of the plasma jet setup. A 500 Ω ballast resistor *R_b_* was used to limit the current, and the plasma current was obtained by measuring the voltage across a 10 Ω resistor *R_i_*. When the applied electric pulsed voltage reaches the breakdown threshold of the working gas, ionization collisions initiated by free electrons induce an electron avalanche, resulting in streamers (also known as ionization waves) and transitioning the previously insulating gas into a plasma phase. The streamers initially move along the nozzle wall as surface ionization waves (SIWs). Upon exiting the nozzle, plasma streamers continue to propagate along a path predetermined by the annular jet flow of the low ionization energy working gas, ionizing and exciting the neutral gas ahead of them until they reach the substrate. (b) Applied voltage and plasma current measured for a pulsed plasma voltage of -2.5 kV at 20 kHz.


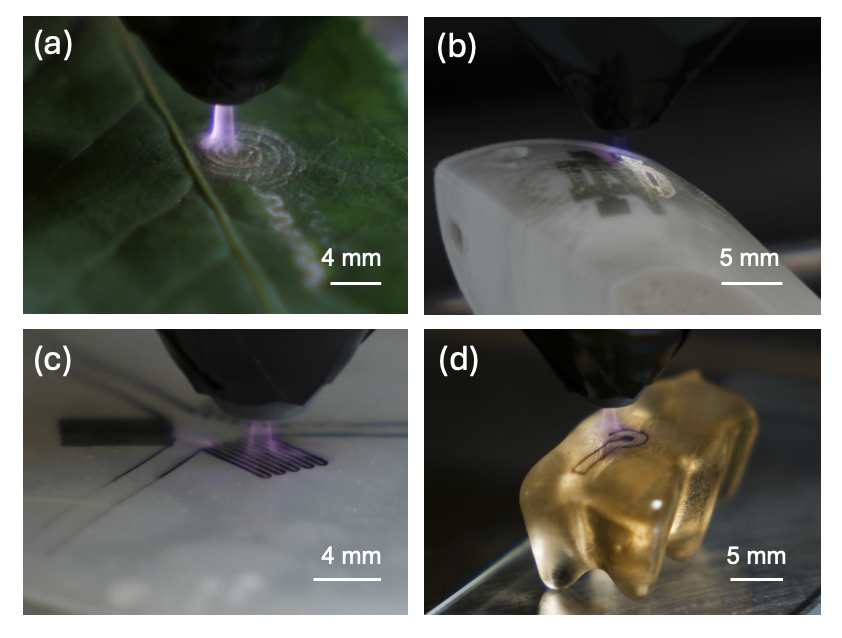


Figure S2: Co-Jet printing process on (a) a leaf, (b) a representative 3D printed polylactic acid (PLA) artifact, (c) an Ecoflex^TM^ silicone rubber membrane, and (d) gelatin (a gummy bear).


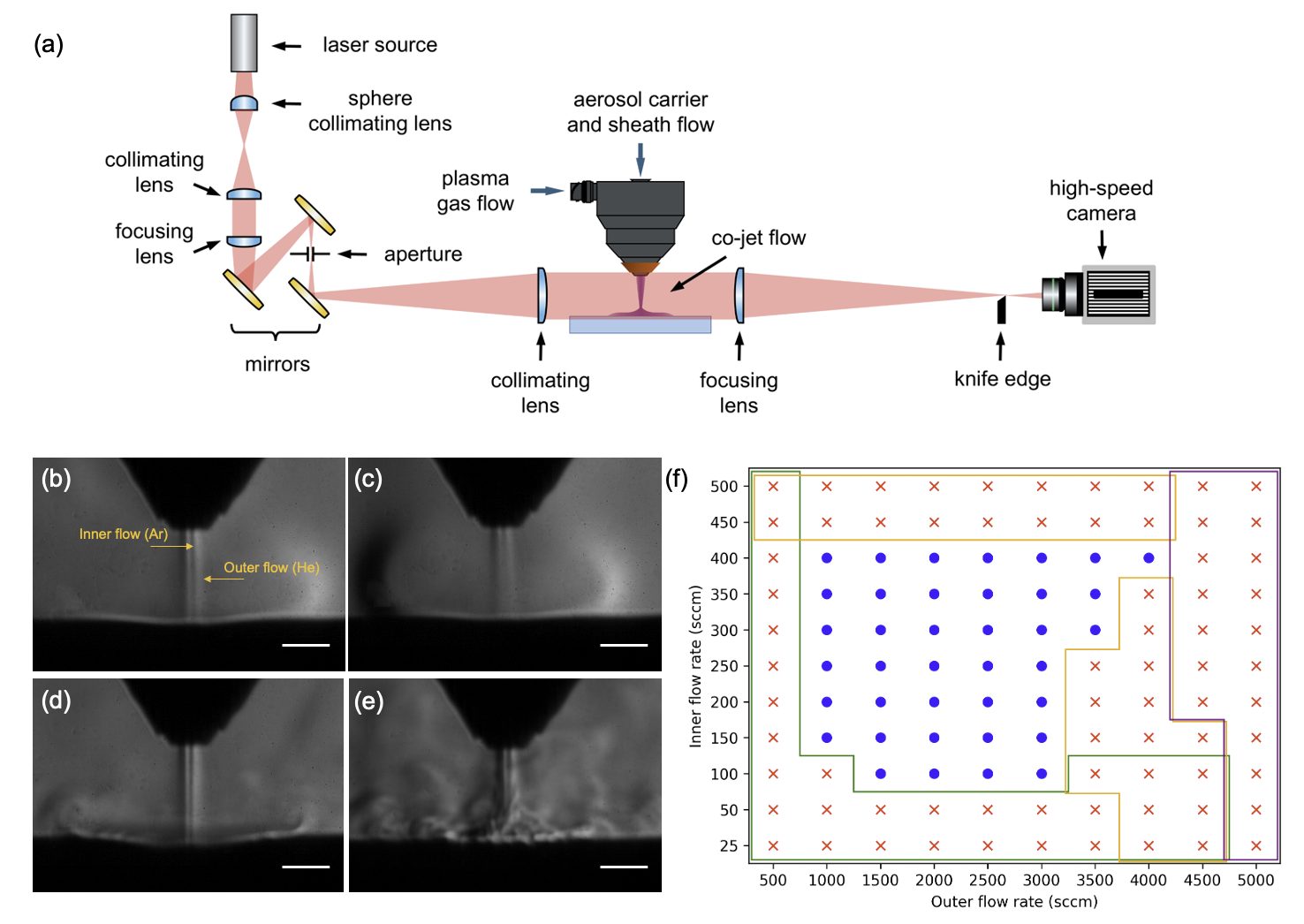


Figure S3: Schlieren imaging visualization of the co-jet flow existing the nozzle employed in this work. (a) Schematic of the Schlieren imaging setup utilized in this work. The nozzle was positioned 10 mm above a glass substrate. (b) Schlieren image of the co-jet flow with an inner flow of 150 sccm and an outer flow of 2000 sccm. The co-jet remains laminar both before and after reaching the glass substrate. (c) Schlieren image of the co-jet with an inner flow rate of 25 sccm and an outer flow of 2000 sccm. The inner flow starts to mix with the outer flow shortly after existing the nozzle outlet due to the stronger shear at the interface between the two jets, constraining the delivery of aerosol droplets over larger standoff distances. (d) Schlieren image of the co-jet with an inner flow rate of 500 sccm and an outer flow of 2000 sccm. While maintaining a laminar co-jet in flight, this gas combination results in instability upon impinging on the substrate. (e) Schlieren image of the co-jet with an inner flow of 200 sccm and an outer flow of 5000 sccm. The co-jet transitions to turbulence before reaching the substrate due to the excessively strong shear between the outer flow and the static ambient air. (f) The operating window of inner and outer flow. The blue dots denote stable laminar flow, while the red crosses indicate non-ideal flow behavior. Specifically, the crosses within the green frame represent early mixing of flows prior to impinging on the substrate, those within the orange frames signify the instability developing after impinging on the substrate, and the crosses within the purple frame correspond to turbulent flow behavior.


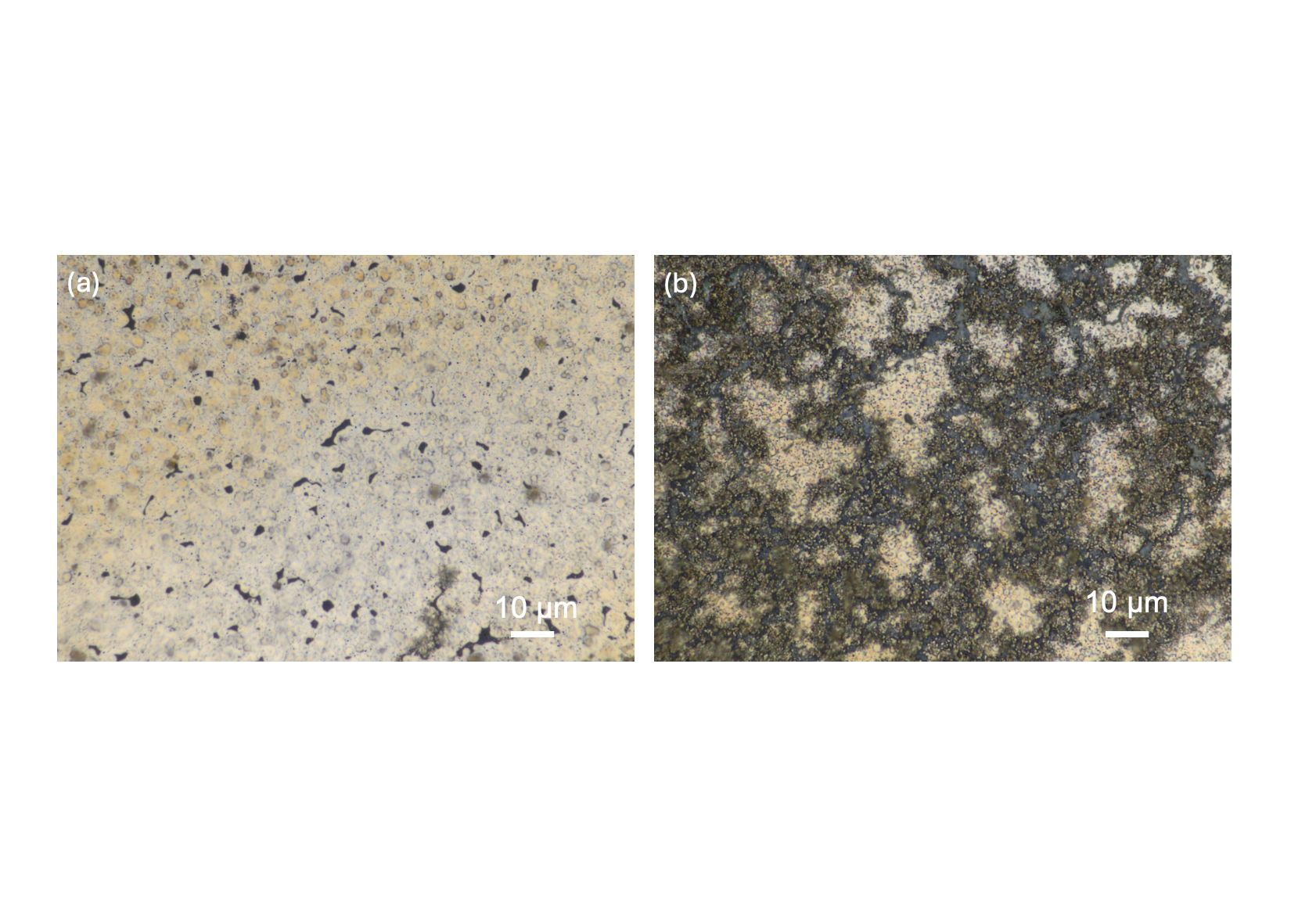


Figure S4: microscope images of (a) the printed film with holes under low CGFR = 7.3 sccm and (b) the corresponding cracks after plasma jet sintering under low CGFR = 7.3 sccm, plasma gas flow rate = 1500 sccm, and plasma applied voltage = -2.7 kV.


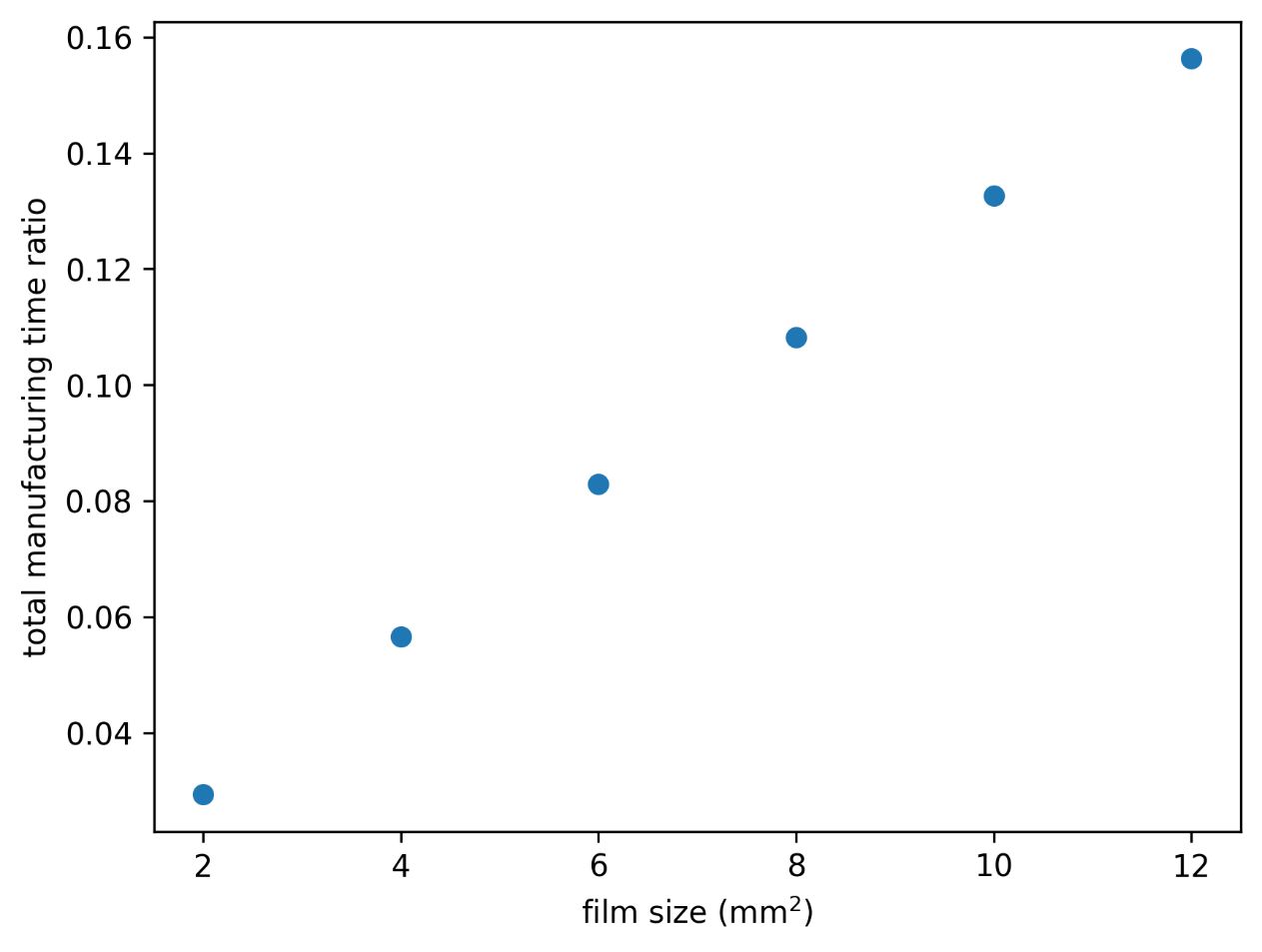


Figure S5: relationship between total manufacturing time ratio (conventional manufacturing time divided by CJP manufacturing time) and film size. Three layers are printed for CJP samples, while two layers are printed for conventional manufacturing samples. The infill pattern line spacing was set to 0.04 μm, and the printing speed was 1 mm/s.

The total manufacturing time ratio $\alpha$ is defined as

$\alpha=\frac{t_{CJP}}{t_{AJP}+t_{sinter}}$ *(S1)*

where $t_{CJP}$ is the total manufacturing time of a film using CJP method with silver nanoparticle ink, $T_{AJP}$ is the printing time of a film using conventional AJP, and $T_{sinter}$ is the typical time required for post-printing thermal sintering of a film.


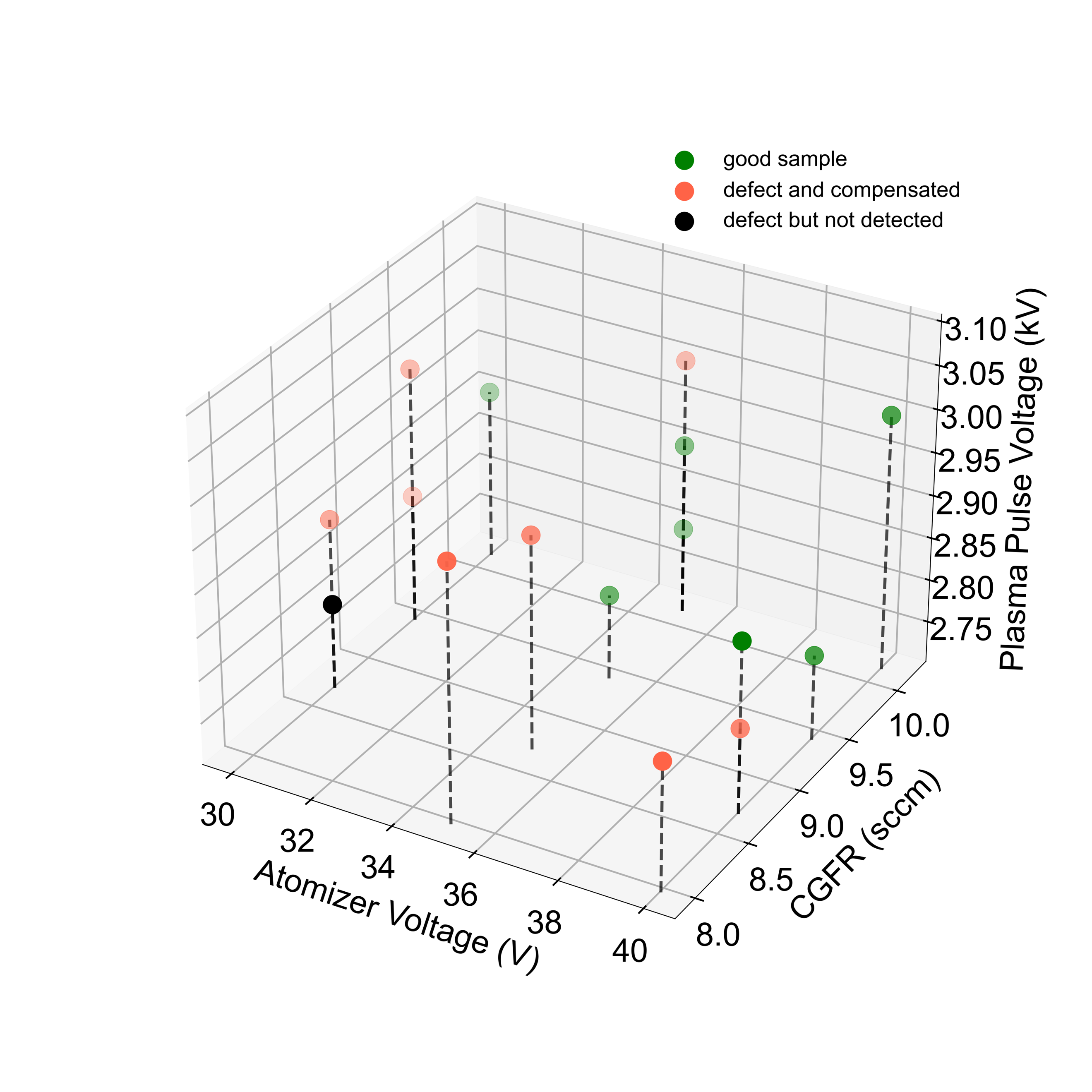


Figure S6: distribution of 16 samples printed with randomly generated printing parameters and their online detection and compensation results.


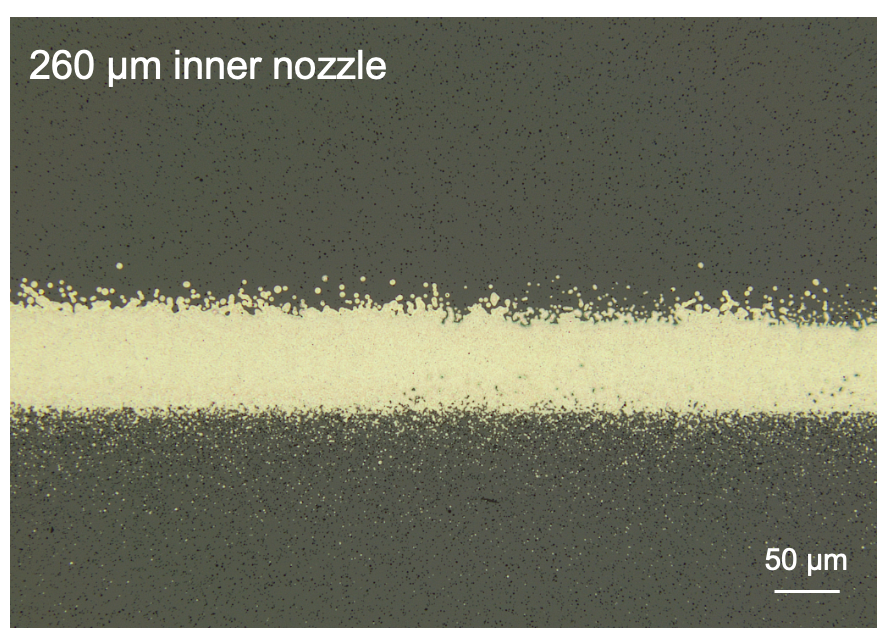


Figure S7: line pattern printed using CJP with a 260 inner nozzle. Different gas combinations were optimized for the smaller nozzle outlet, with the sheath gas flow rate set to 50 sccm, the carrier gas flow rate to 8 sccm, and the plasma gas flow rate to 1300 sccm respectively.


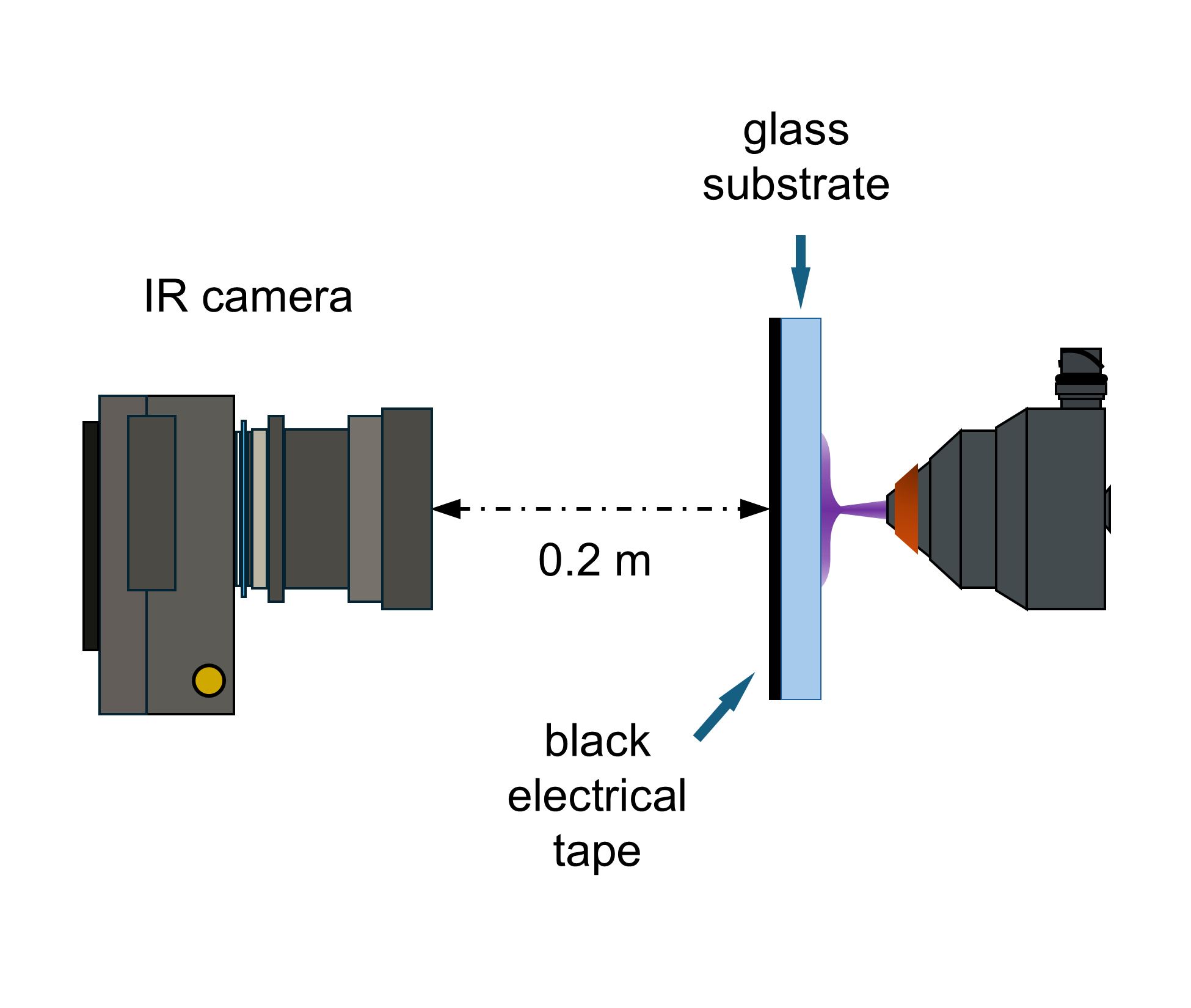


Figure S8: Schematic of IR camera measurement of plasma sintering temperature.


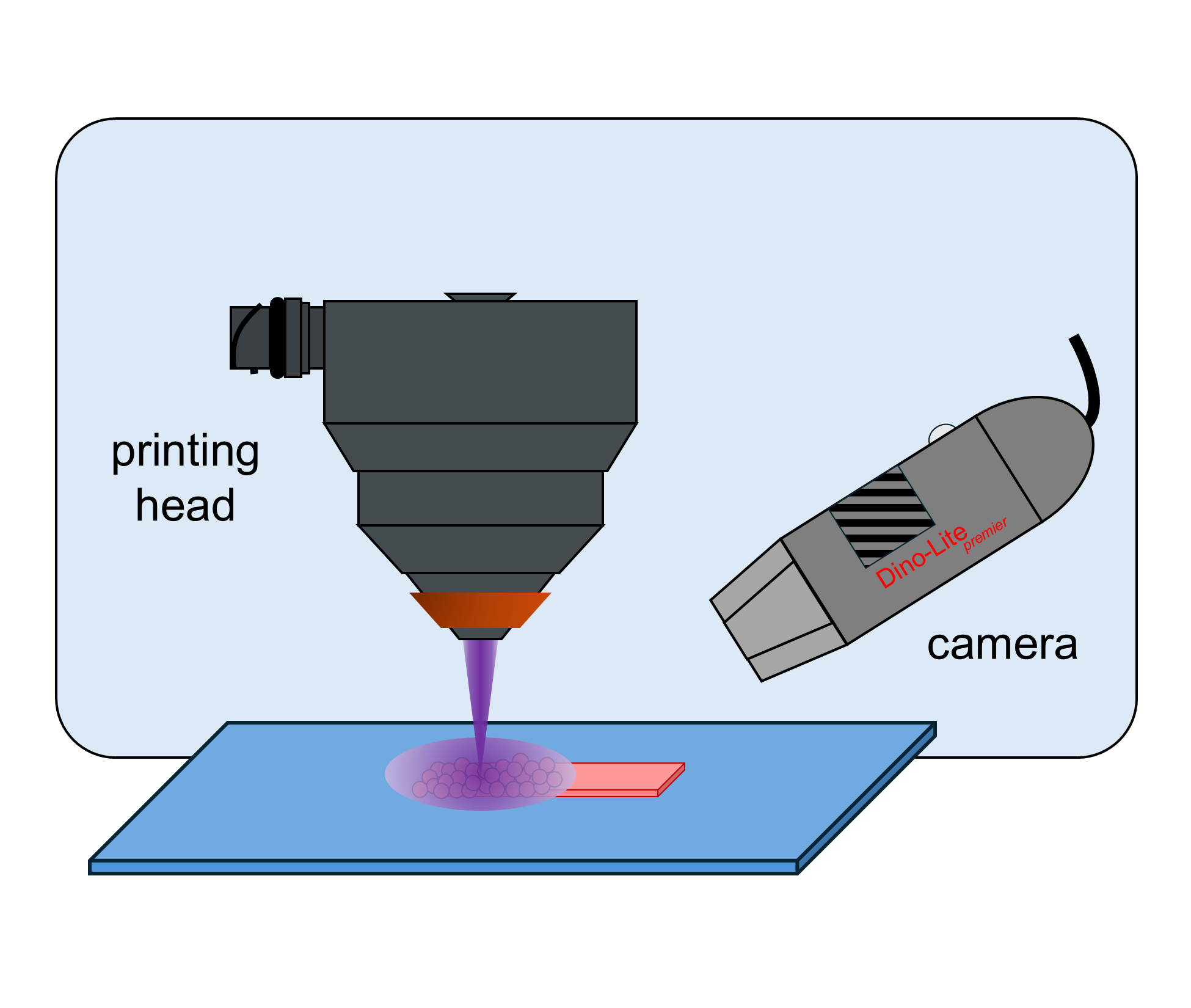


Figure S9: Schematic of the *in-situ* inspection setup.

Table S1: Average film thickness fabricated with varies printing and sintering parameters.

| Sheath gas flow rate (sccm) | Carrier gas flow rate (sccm) | Plasma flow rate (sccm) | Plasma pulse voltage (kV) | Number of layers | Average thickness (μm, *n* = 3) |
| --- | --- | --- | --- | --- | --- |
| 120 | 8.7 | 1885 | -2.3 | 3 | 1.75 |
| 120 | 8.7 | 1885 | -2.4 | 3 | 1.90 |
| 120 | 8.7 | 1885 | -2.5 | 3 | 1.65 |
| 120 | 8.7 | 1885 | -2.6 | 3 | 1.84 |
| 120 | 8.7 | 1885 | -2.7 | 3 | 1.80 |
| 120 | 8.7 | 1885 | -2.8 | 3 | 1.80 |
| 120 | 8.7 | 1885 | -2.9 | 3 | 1.73 |
| 120 | 8.7 | 1305 | -2.7 | 3 | 1.82 |
| 120 | 8.7 | 2465 | -2.7 | 3 | 1.53 |
| 120 | 8.7 | 3045 | -2.7 | 3 | 1.43 |
| 120 | 10.2 | 1885 | -2.9 | 3 | 2.00 |
| 120 | 10.9 | 1885 | -2.9 | 3 | 2.76 |
| 120 | 11.6 | 1885 | -2.9 | 3 | 3.40 |
| 120 | 12.3 | 1885 | -2.9 | 3 | 4.30 |
